# Gene Expression Differences among Three *Neurospora* Species Reveal Genes Required for Sexual Reproduction in *Neurospora crassa*

**DOI:** 10.1371/journal.pone.0110398

**Published:** 2014-10-20

**Authors:** Nina A. Lehr, Zheng Wang, Ning Li, David A. Hewitt, Francesc López-Giráldez, Frances Trail, Jeffrey P. Townsend

**Affiliations:** 1 Department of Ecology and Evolutionary Biology, Yale University, New Haven, Connecticut, United States of America; 2 Department of Biostatistics, Yale University, New Haven, Connecticut, United States of America; 3 Department of Botany, Academy of Natural Sciences, Philadelphia, Pennsylvania, United States of America; 4 Wagner Free Institute of Science, Philadelphia, Pennsylvania, United States of America; 5 Department of Plant Biology, Michigan State University, East Lansing, Michigan, United States of America; 6 Department of Plant, Soil and Microbial Sciences, Michigan State University, East Lansing, Michigan, United States of America; 7 Program in Computational Biology and Bioinformatics, Yale University, New Haven, Connecticut, United States of America; 8 Program in Microbiology, Yale University, New Haven, Connecticut, United States of America; Georg-August-University of Göttingen Institute of Microbiology & Genetics, Germany

## Abstract

Many fungi form complex three-dimensional fruiting bodies, within which the meiotic machinery for sexual spore production has been considered to be largely conserved over evolutionary time. Indeed, much of what we know about meiosis in plant and animal taxa has been deeply informed by studies of meiosis in *Saccharomyces* and *Neurospora*. Nevertheless, the genetic basis of fruiting body development and its regulation in relation to meiosis in fungi is barely known, even within the best studied multicellular fungal model *Neurospora crassa*. We characterized morphological development and genome-wide transcriptomics in the closely related species *Neurospora crassa, Neurospora tetrasperma,* and *Neurospora discreta*, across eight stages of sexual development. Despite diverse life histories within the genus, all three species produce vase-shaped perithecia. Transcriptome sequencing provided gene expression levels of orthologous genes among all three species. Expression of key meiosis genes and sporulation genes corresponded to known phenotypic and developmental differences among these *Neurospora* species during sexual development. We assembled a list of genes putatively relevant to the recent evolution of fruiting body development by sorting genes whose relative expression across developmental stages increased more in *N. crassa* relative to the other species. Then, in *N. crassa*, we characterized the phenotypes of fruiting bodies arising from crosses of homozygous knockout strains of the top genes. Eight *N. crassa* genes were found to be critical for the successful formation of perithecia. The absence of these genes in these crosses resulted in either no perithecium formation or in arrested development at an early stage. Our results provide insight into the genetic basis of *Neurospora* sexual reproduction, which is also of great importance with regard to other multicellular ascomycetes, including perithecium-forming pathogens, such as *Claviceps purpurea*, *Ophiostoma ulmi,* and *Glomerella graminicola*.

## Introduction

Many ascomycete fungi sexually reproduce by forming three-dimensional fruiting bodies that produce their sexual spores (ascospores) in sacs called asci. The genetics of fruit-body development in the filamentous fungi has been studied extensively in several fungi including *Aspergillus nidulans*, *Neurospora crassa*, *Sordaria macrospora, Fusarium graminearum,* and *Podospora anserina* [1]–[9]. Comparative genomics analyses suggest that meiosis machinery and ascospore production are generally conserved within ascomycetes [10]–[17]. Indeed, much of what we know about meiosis in plant and animal taxa has been deeply informed by studies of meiosis in *Saccharomyces* and, historically, *Neurospora* [18], [19]. Although some genes involved in fungal fruiting body development have been identified by mutagenesis screens and characterized, the comparative genetic basis for the sexual cycle of these organisms in terms of interactions among gene networks has not been little explored [7], [20]. Expression and comparative transcriptomics studies between *N. crassa* and *N. tetrasperma* have revealed key genes involved in asexual development and mating behaviors in these fungi [21], [22]. However, inference and comparison of regulatory pathways for sexual development across these species has been difficult to achieve, partly due to the complex environmental stimuli relevant to sexual development and partly due to a lack of molecular detail regarding relevant gene interactions across sexual development. *Neurospora* species represent attractive models for elucidating the regulation of fruiting body development by transcriptional profiling and functional analysis due to their simple nutritional requirements, fast vegetative growth, and clearly recognizable stages during sexual development [23], [24]. The most studied species is *N. crassa* [25], particularly well-known for its use in the original “one gene-one enzyme” experiments by Beadle and Tatum, for its eight-spored ordered asci which enabled centromere mapping of mutants, and for its experimentally tractable and intensively-studied genetic network for circadian rhythm [26], [27].

The life histories of *Neurospora* spp. span the most common sexual strategies in the fungal kingdom, i.e. heterothallism (self-incompatibility with distinct mating types), pseudohomothallism (self-compatibility in which paired mating types coexist in one mycelium), and homothallism (self-compatibility regardless of mating type). Initiation of sexual reproduction is regulated by mating type genes and leads to cell fusion, nuclear pairing, nuclear fusion, meiosis, and the production of haploid ascospores. The determinant sequences for mating type, *mat A* and *mat a*, are at the same genetic locus in a given species but exhibit little to no similarity in nucleotide sequence; that is, they represent idiomorphs rather than alleles [28]. Heterothallic *Neurospora* species such as *N. crassa* and *N. discreta* [23] possess a bipolar mating system, and two strains with opposite mating types, *mat A* and *mat a*, must cross to initiate sexual development. Either mating type can produce female structures (protoperithecia with a trichogyne) as well as male reproductive structures (conidia) [29], [30]. Pseudohomothallic species such as *N. tetrasperma* are generally self-compatible, even though they also require both mating types (*A* and *a*) to reproduce. In *N. tetrasperma*, both mating types are generally found within a single individual, although strains containing a single mating type exist in nature and can be isolated in lab. For *N. tetrasperma*, *mat A* and *mat a* nuclei associated in pairs in four heterokaryotic spores, packaged within an ascus for discharge [31]. Despite mating behavior differences that distinguish *N. tetrasperma* from *N. crassa* and *N. discreta*, phylogenetically *N. tetrasperma* and *N. crassa* are closely related and share the most recent common ancestor with *N. discreta* [32].

The genetic basis of sexual development has been the subject of many investigations, with unresolved controversy arising about the ancestral state of the life style of fungal sexual reproduction, heterothallism or homothallism, and its underlying genetic mechanism(s) [33]–[40]. For example, the heterothallic life style has been suggested to be ancestral within *Neurospora* [31], because the pseudohomothallic *N. tetrasperma* does not require a mating partner, but does require both *mat* idiomorphs to complete the sexual cycle. However, function of *mat* genes in *Neurospora* sexual development is not well understood, except their roles in heterothallic species such as *N. discreta* and *N. crassa* as regulators of pheromone expression to direct hyphal growth and fusion [4], [41]. For heterothallic species as in *N. crassa*, the trichogynes, which originate from protoperithecia, change their direction of growth to approach conidia of the opposite mating type and fuse with them. Plasmogamy is followed by development of the perithecium, and later ascal development, karyogamy, and then formation of ascospores [42]–[44]. Two haploid nuclei of opposite mating type fuse in a young ascus resulting in a diploid zygote nucleus that immediately undergoes meioses, followed by a postmeiotic mitosis. The mature ascus delimits eight linearly arranged ascospores, each containing a single nucleus. After mitosis the ascospores become binucleate, gradually grow to their full size, and become pigmented.

Genomes of multiple species of *Neurospora* have been sequenced, and comparative genomic analyses have been focused on *N. crassa* and its closely related species [3], [6], [45]. Here we reveal candidate genes involved in fungal development and in the evolution of perithecia by comparisons of the gene expression levels within and across three *Neurospora* species. We assayed for large-scale differences in morphology and the transcriptomic landscape over the time course of sexual development, identifying putative genes involved in sexual development by comparative gene expression profiling. We also tested for knockout phenotypes of selected candidate genes by assaying knockout strains across sexual development for their ability to produce wild type perithecia. Our results provide insights into the links between gene expression and sexual development of *N. crassa* and related species, as well as contributing to our understanding of how fungi reproduce sexually.

## Materials and Methods

### 2.1. Strains and culture conditions

Strains of complementary mating types *mat a* and *mat A* for *N. crassa* (FGSC4200, FGSC2489), *N. tetrasperma* (FGSC2509, FGSC2508) and *N. discreta* (FGSC8578, FGSC8579) were obtained from the Fungal Genetics Stock Center (FGSC) [46]. The strains were grown on Carrot Agar (CA), made as previously described [47]. The CA petri dish was covered with a cellophane membrane (Fisher Scientific Company) and plugs of agar with strains were deposited on the membrane and incubated at 26°C under constant artificial light from several Ecolux bulbs (F17T8.SP41-ECO, General Electric Company), which provided a net intensity of 14 µMol/m^2^ S at the media surface. Conidia from the *mat a* strain on CA were collected and suspended in 2.5% Tween 60 (10^5^–10^6^ conidia/ml). Cultures of the *mat A* strain on CA were examined using a stereomicroscope for the formation of protoperithecia in 5–7 days, and areas with evenly distributed protoperithecia of a common size were delineated with a marker on the bottom of the plate to be harvested for stage-specific transcriptomics.

Crosses were performed by applying 2 ml of the suspension of *mat a* conidia in 2.5% Tween 60 (10^5^–10^6^ conidia/ml) to the surface of the *mat A* protoperithecia plates, at which point considerable disturbance to surface hyphae and other fungal tissues was unavoidable. Sexual development was monitored with a stereomicroscope until fully developed perithecia appeared [48]. Fungal material was harvested by scraping the surface with a razor blade in the areas, where protoperithecia or young perithecia similar in size were densely aggregated, right before the crossing and at 2, 24, 48 h after crossing. Sets of individual perithecia of similar morphological development were picked at 72, 96, and 144 h after crossing. For transcriptomic analysis, all tissues and perithecia were immediately and rapidly frozen in liquid nitrogen as they were sampled, then stored at −80°C.

### 2.2. Fixation and microscopy

Perithecium development was monitored for all three *Neurospora* species with a stereomicroscope over the time course of the sexual development. Cultures and crossings were performed as described in 2.1. Pieces of cellophane membrane (about 4 mm×2 mm) carrying 5–20 perithecia of similar size were cut from cultures and fixed in 1.5% formaldehyde and 0.025 M phosphate buffer for at least 48 h. The samples were embedded in resin and prepared for light microscopy as previously described [48]. Briefly, resin blocks were sectioned to a thickness of 1 to 2 µm using a glass knife and stained with 1% toluidine blue. A Leica DM LB microscope (Leica Microsystem Gmbh, Wetzlar, Germany) was used to capture images using a Zeiss AxioCam MRc color camera and AxioVision 4.8.2 (Göttingen, Germany). Image processing and annotation were performed using Adobe Photoshop CS3 (San Jose, CA).

To compare mature perithecia, scanning electron microscopy (SEM) was performed. Perithecia were collected, including the cellophane membrane they were growing on, and fixed immediately in 0.1 M sodium cacodylate buffer (pH 7.2) containing 2% glutaraldehyde over night. The samples were then washed with 0.1 M sodium cacodylate buffer, postfixed with 1% osmium tetroxide for 1 h, washed with distilled water, and dehydrated in an increasing series of ethanol. Then, all samples were critical point dried, mounted on an SEM stub, and sputter coated with gold. The samples were examined with a Scanning Electron Microscope (ISI SS-40).

### 2.3. RNA extraction, cDNA preparation, and transcriptomic sequencing

RNA was isolated from homogenized mycelia with TRI REAGENT (Invitrogen) and RNAeasy Kit (Qiagen) following protocol described by Clark et al. [49] and mRNA was purified using Dynabeads oligo(dT) magnetic separation (Invitrogen). The cDNA libraries for RNA sequencing were prepared according to the Illumina mRNA Sequencing Sample Preparation Guide. In brief, triplicates were prepared for each time point and pooled. Then, mRNA was purified from total RNA and 100 ng (9 µl) were fragmented with 10X fragmentation buffer (Ambion AM8740) and incubated at 70°C for 5 min prior to adding 1 ul stop buffer (Ambion). Fragmented mRNA was precipitated using 100% ethanol with glycogen (Ambion) at −80°C. Random hexamers (N_6_, Invitrogen) were added to prime reverse transcription of the first strand cDNA separately for each sample, and to recover the second strand cDNA for all samples. After the ligation of standard adapters for Illumina sequencing, all samples were separated on a 2% low melting point agarose gel and processed cDNA fragments of lengths between 200 and 400 bp were selected by gel extraction and purified with Qiaquick gel extraction kit (Qiagen). The quantity of the samples was increased by a PCR using Pfx DNA polymerase (Invitrogen), and 15 cycles of PCR, each cycle comprising 98°C for 10 s, 65°C for 30 s, and 68°C for 30 s. The quantity and quality of the purified PCR products were checked at the Yale Center for Genome Analysis prior to sequencing. Single-end 35 bp reads of N_6_-primed preparations were separately sequenced, each on eight lanes of an Illumina Genome Analyzer (Yale Center for Genome Analysis).

### 2.4. Data acquisition and analysis

The libraries were run on eight lanes of an Illumina Genome Analyzer, generating an average of 28 million single-end reads of 36 nucleotides each. Since transcriptomic tags also contain sequences that span exon junctions, the program Tophat v1.1.14 [50] was used to perform spliced alignments of the tags against the *N. crassa* OR74A genome (NC10; [51]), and those of *N. tetrasperma* FGSC 2508 (v2) and *N. discreta* FGSC 8579 (v1) obtained from JGI Genome Portal [52]. We scored results only for tags that mapped to a single unique location in the genome (–max-multihits option was set to 1) with less than three mismatches (–splice-mismatches option was set to 2). We used the default settings for all other Tophat options. We tallied tags aligning to exons of genes with the program HTSeq v0.4.5p6 (Unpublished; http://www-huber.embl.de/users/anders/HTSeq/doc/) and the gene structure annotation file for the reference genome. LOX v1.4 [53] was applied to the tallies for each sample for each gene to estimate gene expression levels and credible intervals across developmental stages. LOX provided relative gene expression levels standardized by the lowest sample, with credible intervals, for all three species (Table S1).

Instead of using simple sequence similarity for homolog identification, we applied a phylogenetic approach that is reliable in calling homologs among closely related genomes, with a cost of power to identify potential homologs for recently evolved gene families and genes experiencing multiple duplication events in their evolutionary history. A total of 2352 orthologous genes were selected by the BranchClust method [54] for *N. crassa*, *N. tetrasperma* and *N. discreta*. BranchClust uses the Reciprocal Best Blast hit method and phylogenetic trees to select putatively orthologous genes using a default threshold e-value of 10^−4^; we only choose the complete families in those three species. Transcriptomic sequencing revealed expression levels for all orthologous gene triads across sexual development (Table S2). Data is also deposited as accession 239 at the Filamentous Fungal Gene Expression Database (FFGED; [55]) and as accession GSE41484 for *N. crassa* [56], GSE60256 for *N. tetrasperma*, and GSE60255 for *N. discreta* at the National Center for Biotechnology Information Gene Expression Omnibus (GEO; http://www.ncbi.nlm.nih.gov/geo). More orthologs can be called among these fungi using BLAST approaches, and we also identified more single copy ortholog for these species using a BLAST-based method. However, from our other evolutionary study targeting several gene families with these species, we found that phylogeny-based ortholog calls by BranchClust were more robust and reliable.

A comparative heat map was constructed based on the level of expression of each gene for all three species over the entire time course of sexual development. Gene expression levels for each ortholog were normalized row-by-row by subtraction of the mean and division by the standard deviation. To cluster observed gene expression hierarchically, rows only were clustered by iteratively agglomerating the most similar two gene expression profiles and by averaging the agglomeration for the next iteration (Unweighted Pair Group Method with Arithmetic mean, UPGMA) within which similarity was assessed by the Pearson correlation coefficient of expression between genes. The Functional Catalogue (FunCat: [57]; http://mips.helmholtz-muenchen.de/proj/funcatDB/) annotation scheme was used to group genes according to their functions. The statistically significant overrepresentation of gene groups in functional categories relative to the whole genome was determined by a hypergeometric distribution *P* value calculation, facilitated by the MIPS FunCat online web application.

### 2.5. Comparative gene expression analysis of *N. crassa*, *N. tetrasperma,* and *N. discreta*

The LOX estimates across developmental stages for each orthologous gene for each species were assembled to compare gene expression levels across development of *N. crassa*, *N. tetrasperma*, and *N. discreta*. For each developmental stage, two values were calculated using the upper bound of the 95% confidence interval (CI) or the lower bound of the 95% CI from LOX. If the expression of a gene in species 1 was higher than species 2, we calculated the difference between the lower bound of expression in species 1 and the upper bound of expression in species 2. Conversely, if the expression of a gene in species 2 was higher than species 1, we calculated the difference between the lower bound of species 2 and the upper bound of species 1. These differences constitute those of which we can be highly confident. *N. crassa* was compared with *N. tetrasperma*, *N. crassa* with *N. discreta*, and lastly *N. tetrasperma* with *N. discreta*. The calculation described above was performed for all time points (2, 24, 48, 72, 96 and 144 h) for each gene. Finally, for each comparison, all genes were prioritized according to magnitude of difference in a descending order and the first 130 genes with the biggest changes across all time points and common to all comparisons were subsequently selected for knockout and phenotyping. The data for the comparative gene expression analysis can be found in the Tables S3–S5.

### 2.6. Assessing phenotypes of knock out mutants

Knockout strains for the top 130 candidate genes were compared to wild-type (WT) strains and screened for defects in fruiting body formation. Knockouts of candidate genes were obtained for both mating types in *Neurospora crassa* from the Fungal Genetics Stock Center (FGSC). These knockouts had been preliminarily assayed for phenotypes by several high-throughput screens as part of a *Neurospora* knockout project [58], [59], and in that project had not exhibited a mutant phenotype in asexual growth or development. We performed a detailed, controlled screen crossing the two mating types of each deletion strain, cultured on CA in triplicate. The mat A cultures on CA were fertilized with conidia from the mat a strain. Perithecium formation was monitored with stereo- and light microscopy (Nikon Diaphot 300) for the presence and for the orientation of a single beak, black coloration and the typical vase-shape. Mature perithecia were examined in squash mounts for the presence of asci with normal pores, ascospores with normal shapes and numbers, and normal paraphyses. Normal spore development and ascus firing were examined by checking the lid of the Petri dish for black ascospores.

### 2.7 Cosegregation experiments of knockout mutants showing a phenotype different from the wild type

Knockouts of candidate genes obtained from FGSC were produced using a high-throughput gene deletion strategy in *N. crassa* strains with deletion mutations of *mus-51* and/or *mus-52*, mutations required for nonhomologous end-joining DNA repair. [58], [59]. A high rate of spontaneous mutations has been observed in in *Δmus-51* and/or *Δmus-52* strains, and cosegregation experiments were used to demonstrate that the intended knockout deletion was responsible for the mutant phenotype [60]. A hygromycin resistance cassette at the location of the deletion mutation provides a selectable marker. The KO strains showing phenotypes in sexual development were crossed with wild-type strains (FGSC2489 *mat A* or FGSC4200 *mat a*). Individual ascospore progeny were isolated for resistance to hygromycin. Their phenotypes were then examined on SCM medium. Cosegregation of hygromycin resistance and the observed phenotype is necessary evidence that the observed phenotype was result of the deletion of the specified gene.

## Results

### 3.1 Perithecium development in *N. crassa*, *N. tetrasperma*, and *N. discreta* on CA follows a common time course

**Figure 1:**
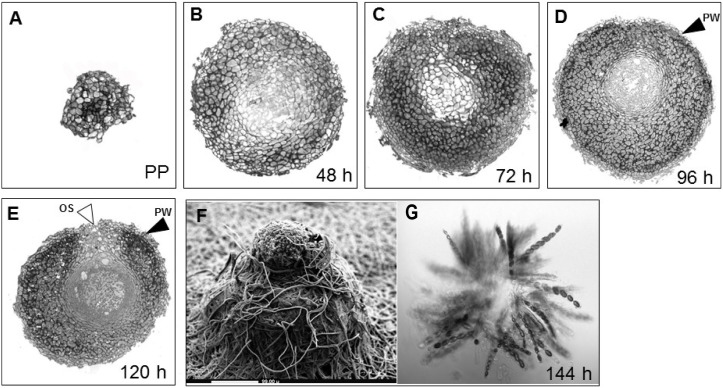
Sexual development of *N. crassa*. Cross sections of developing perithecia, from A) the protoperithecium (PP) through a time course of (B) 48 h, (C) 72 h, (D) 96 h, and (E) 120 h after fertilization. These images illustrate the development of several cell layers within the fruiting body such as the perithecium wall (PW, black arrowheads), composed of thick-walled cells, as well as the initial stages of formation of asci and ascospores and the ostiole (OS, white arrowheads) through which spores are released in a later stage. After (F) 144 h, the perithecium including its beak is fully developed, as shown by scanning electron microscopy (SEM). (G) A squash mount of a mature fruiting body, showing asci and ascospores.

**Figure 2:**
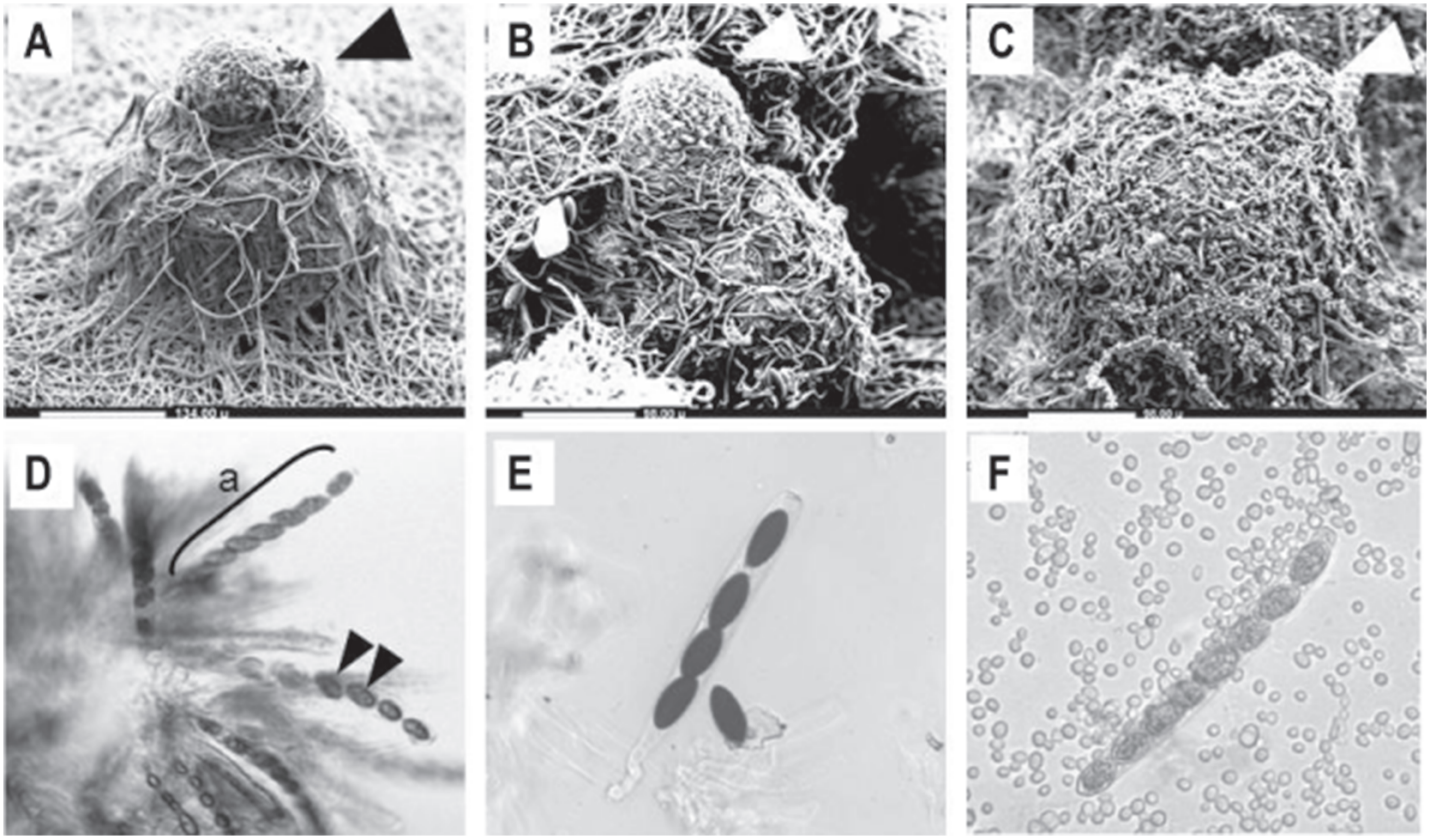
Key morphological characters of *N. crassa, N. tetrasperma* and *N. discreta* 144 h after crossing. Scanning electron micrographs, in which arrowheads indicate the perithecial beaks, of A) *N. crassa* (bar: 134 µm), B) *N. tetrasperma* (bar: 98 µm) and C) *N. discreta* (bar: 98 µm), and light micrographs of squash mounts of (D) *N. crassa* (a: ascus), (E) *N. tetrasperma*, and (F) *N. discreta* (with conidia). At 144 h, some spores are not fully mature.

To monitor the sexual development of all three *Neurospora* species, we crossed wild-type strains of both mating partners of each *Neurospora* species. The observed perithecial development of *N. crassa, N. tetrasperma*, and *N. discreta* aligned with the common time course known for these species [56]. We observed that during perithecial development, grayish to yellowish gray protoperithecia darkened, then blackened once mature perithecia had formed, indicating the biosynthesis of melanin in the time course of perithecial development (Fig. 1). As expected, we also identified the fading of the orange pigmentation in the colony across the time course of sexual development, indicating a reduction in the asexual phase of the life cycle. Additionally, we found no obvious tissue differentiation between protoperithecia and perithecia within 24 h after crossing, except for a slight increase in size and a slight darkening in colour, indicating the biosynthesis of melanin as part of the successful fruiting body formation. Furthermore, the centrum parenchyma of thin-walled cells expanded with increasing perithecial size and differentiated filamentous structures and croziers formed after 48 h to 72 h. Asci containing developing ascospores were visible after 96 h, along with some narrow paraphyses. From 120 h to 144 h post crossing, a beak formed at the apex of the perithecium, and scanning electron microscopy of all three species revealed fully developed fruiting bodies with a beak on top of the perithecium (Fig. 2, panels A–C). Furthermore, squash mounts of perithecia 144 h after crossing revealed that the inside of the perithecium contained mature asci with ascospores (Fig. 2, panels D–F). A major phenotypic difference among species was the number of spores produced, which, as expected in *N. tetrasperma* was four, in contrast to eight in *N. crassa* and *N. discreta.* Additionally, *N. discreta* produced abundant conidia, which were visible associated with the perithecia after the sexual cycle has concluded (Fig. 2, panels C and F).

### 3.2. Transcriptional profiling of *N. crassa*, *N. tetrasperma*, and *N. discreta* reveals eight functional categories

**Figure 3:**
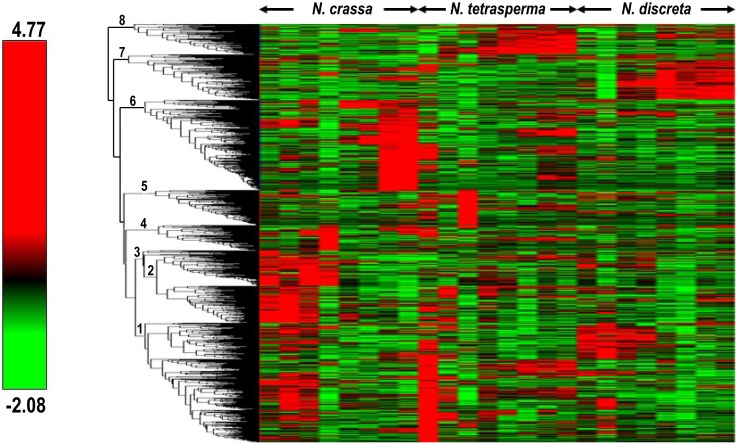
Comparative heat map of *N. crassa* (*N.c*), *N. tetrasperma* (*N.t*) and *N. discreta* (*N.d*) gene expression. Relative gene expression levels of the orthologous genes in all three species inferred by BranchClust, from Before Crossing (BC) to 144 h after crossing, were normalized row-by-row by subtraction of the mean and division by the standard deviation. Rows were hierarchically clustered by average linkage (UPGMA) applied to the Pearson correlation coefficient. Clusters 1–8 were analyzed for their function with FunCat. The scale of mean-centered relative gene expression ranges from −2.08–4.79, as indicated with the scale bar.

To determine the gene expression of all three *Neurospora* species during the time course of sexual development, we performed Illumina next generation transcriptomic sequencing. We prepared samples by isolation of mRNA and performance of reverse transcription of genomic RNA with random hexamers (N_6_), which were typically consistent in DNA concentration. Single-end 35-base reads of N_6_-primed preparations were sequenced separately, each developmental stage of each species on one of eight lanes of an Illumina Genome Analyzer, at the Yale Center for Genomic Analysis. A total of 2352 single copy orthologous genes were selected by BranchClust [54] for *N. crassa*, *N. tetrasperma* and *N. discreta*. BranchClust uses the Reciprocal Best Blast hit method and phylogenetic trees to select putatively orthologous genes, with default threshold e-value of 10^−4^. Our deep transcriptomic sequencing revealed expression in all three species across sexual development for all of these identified orthologous genes. To compare gene expression levels for these genes, we constructed a comparative heatmap with the Hierarchical Clustering Explorer Software [61] based on the level of expression of each gene for all three species over the entire time course of sexual development (Fig. 3) as described in section 2.4. Transcriptional profiling of *N. crassa*, *N. tetrasperma*, and *N. discreta* revealed eight clusters, which we then analyzed separately for functional category representation.

#### 3.2.1 Among genes highly expressed in early stages of perithecium development, the ortholog set was enriched for genes involved in metabolic processes

Where previously identified, genes were assigned cellular or molecular functions by their Functional Catalogue (FunCat) [57] annotation. The statistical significance of overrepresentation of gene groups in functional categories relative to the whole genome was determined using the hypergeometric distribution, facilitated by the Munich Information Center for Protein Sequences (MIPS) FunCat online web application. Eight major clusters were identified based on expression patterns across sexual development for the identified orthologs. In cluster 1 (Fig. 3), 61 of the 671 proteins were unclassified. Of the classified proteins we identified, most were involved in metabolism, cellular transport, cell cycle and DNA processing, protein with binding function, cellular communication and transcription.

Genes in cluster 2 were mainly involved in metabolism, cellular transport, transport facilities and transport routes as well as proteins with binding function, while only 16 of the proteins in cluster 2 were not classified. The metabolic genes were primarily involved in nucleotide/nucleoside/nucleobase metabolism, C-compound and carbohydrate metabolism, lipid, fatty acid and isoprenoid metabolism and metabolism of vitamins, cofactors, and prosthetic groups. The cellular transport-related genes were involved in transported compounds (substrates), protein transport, electron transport, vesicular transport such as the Golgi network and vacuolar/lysosomal transport. Genes encoding proteins with binding functions such as nucleic acid binding, metal binding and complex cofactor/cosubstrate binding. In cluster 3, only 19 out of 199 proteins were not classified, while the remaining proteins were mainly involved in metabolism, especially C-compound and carbohydrate metabolism. Genes in cluster 3 also tended to be involved in cellular transport, including cation and heavy metal ion transport as well as proteins with binding functions, e.g. nucleic acid binding, metal binding and RNA binding. Cluster 4 contained 139 proteins, which were involved mainly in metabolism, especially lipid, fatty acid and isoprenoid metabolism, cellular transport, and cell cycle including DNA restriction or modification. Overall, we observed an enrichment of genes involved in metabolic processes in early stages of perithecium development.

#### 3.2.2 In later stages of the perithecium development, the ortholog set was enriched for genes involved in transcription, cell cycle, protein synthesis and cellular transport

Based on Functional Category (FunCat, [57]) annotations, cluster 5 comprises 198 proteins, of which the majority was involved in nucleotide/nucleoside/nucleobase metabolism, nucleotide/nucleoside/nucleobase metabolism and lipid, fatty acid, and isoprenoid metabolism, but also transcription such as RNA synthesis and RNA processing.

Interestingly, in clusters 6–8 there appears to be a species-specific expression in later stages of the development. Proteins of cluster 6 were mainly involved in transcriptional processes such as RNA synthesis, processing and modification, but also, cell cycle and DNA processing, as well as and metabolism. Cluster 7 contained 259 genes involved in transcription, protein binding, cell type differentiation and cellular transport. In cluster 8 only 13 genes were not identified. The cluster mainly comprised proteins involved in cell differentiation, cellular transport and transcription.

While in the early stages of the perithecium development, the fungi were observed to exhibit an enrichment of expressed genes involved in metabolic processes; in later stages of perithecium development, expressed genes from the ortholog set were enriched for genes involved in transcription, cell cycle, protein synthesis and cellular transport.

### 3.3 Comparative gene expression analyses revealed genes crucial for the successful development of fruiting bodies

**Figure 4:**
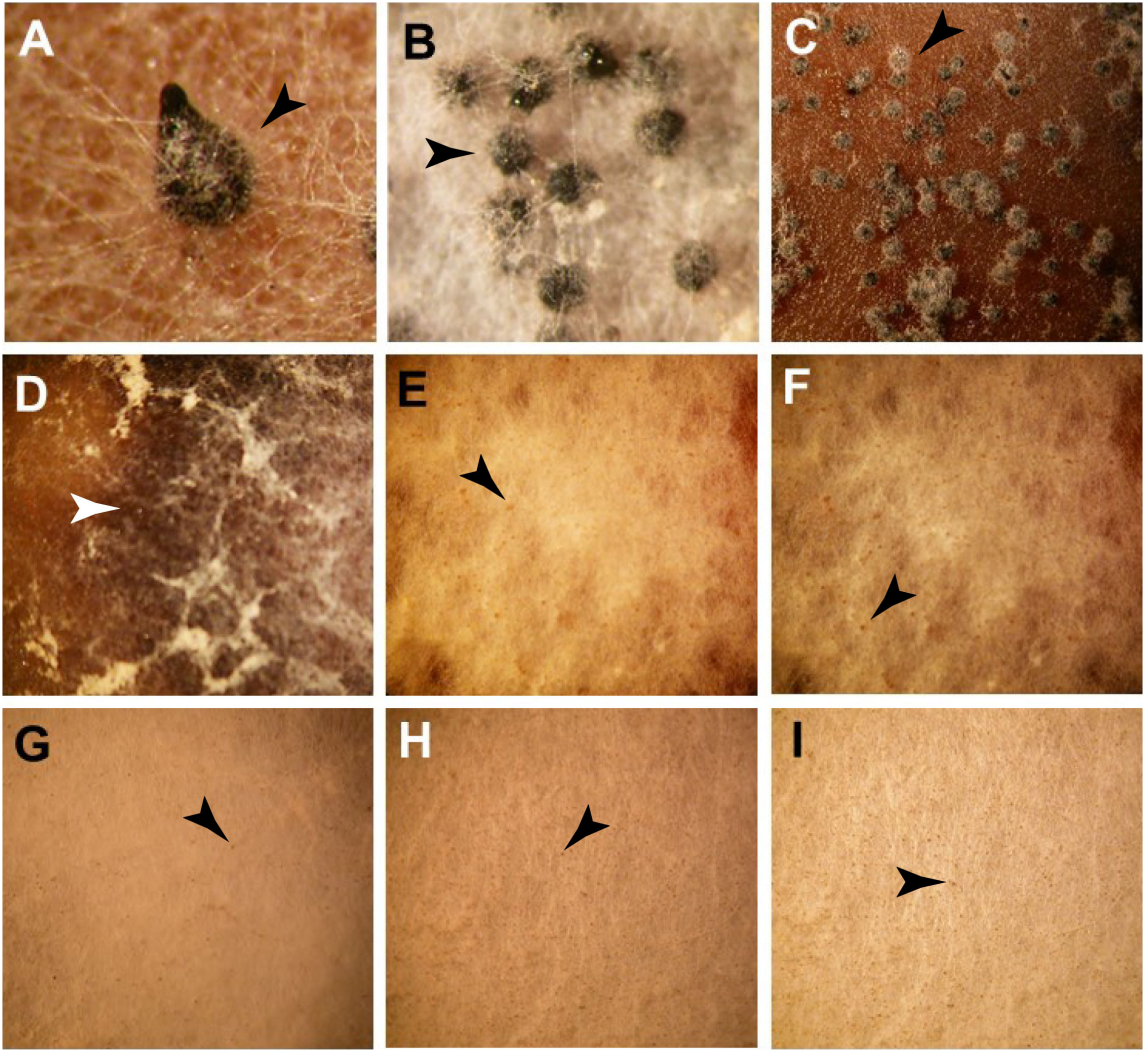
Phenotypes of perithecia from crosses in *N. crassa*. (A) WT control, (B) ΔNCU06316: perithecium development was arrested at early stage equally to 48–72 h, (C) ΔNCU07508: perithecium development was arrested at 48–72 h, (D) ΔNCU06874: no perithecia formed, only protoperithecia, though melanin was released into the medium (white arrow heads), (E) ΔNCU05609: no perithecia formed, only protoperithecia, (F) ΔNCU00175: no perithecia formed, only protoperithecia, (G) ΔNCU00427: no perithecia formed, only protoperithecia. (H) ΔNCU02089: protoperithecia failed to develop into perithecia, and (I) ΔNCU09525: protoperithecia only. Perithecia (large, black) and protoperithecia (small, yellowish-gray to gray) are indicated with black arrow heads.

Crossing of the *N. crassa* WT strains resulted in the successful formation of perithecia with perithecial beak (Fig. 4A). In order to identify genes that are required for the successful development of fruiting bodies, knockouts in *N. crassa* from the whole genome knockout project [58], [59] of the 130 top candidate genes identified through pairwise comparison of orthologous gene expression in *N. crassa*, *N. tetrasperma*, and *N. discreta* were screened for mutant phenotypes in sexual development. Eight mutant phenotypes were observed that affected perithecium formation. Interestingly, development of NCU06874, encoding a HMG box (high mobility group)-containing protein was arrested after formation of protoperithecia on a fluffy white mycelium. Putative functions of the remaining genes that affect fruiting body formation in *N. crassa* were determined by a Blast search [62] with the NCBI Blast server of the translated sequence against all known GenBank non-redundant protein sequences (Table 1). However, the deletion of either gene NCU06316, a putative argonaute siRNA chaperone complex subunit which in fission yeast is required for histone H3 Lys9 (H3-K9) methylation, heterochromatin, assembly and siRNA generation [1], or NCU07508, a putative type-2 protein geranylgeranyltransferase subunit, caused the perithecium development to arrest at an early stage between 48 and 72 h (Fig. 4, panels B and C). In the perithecia arising from these KO strains, we observed no perithecial beak and found that the developing perithecia remained spherical, indicating an arrest in development. Furthermore, the CA of these cultures was stained black along the mating zone, suggesting an increase in melanin biosynthesis (Fig. 4D).

**Table 1:** Putative functions of genes whose deletion impacts fruiting body formation.

ID	Annotation by homology	Max	Total	Query	E-	Max
		score	score	cover	value	iden
NCU06316	argonaute siRNA chaperone complex	246	246	76%	8e–72	37%
	subunit Arb1					
	[*Colletotrichum fioriniae*],					
	XP_007589964.1					
NCU07508	putative type-2 protein	145	145	68%	1e–34	31%
	geranylgeranyltransferase subunit beta					
	protein					
	[*Botryotinia fuckeliana*], EMR87581.1					
NCU06874	HMG box-containing protein	269	269	42%	7e–75	40%
	[*Magnaporthe oryzae*], XP_003718106.1					
NCU05609	proline-rich protein [*Coccidioides*	46.6	46.6	64%	0.020	29%
	*Posadasii*], ACU44647.1					
NCU00175	repetitive proline-rich cell wall protein	153	205	88%	1e–39	44%
	[*Colletotrichum higginsianum*],					
	CCF45612.1					
NCU00427	YjeF_N domain-containing protein	734	734	97%	0.0	53%
	[*Magnaporthe oryzae*],					
	ELQ39075.1					
NCU02089	hypothetical protein SMAC_04986	1276	1276	99%	0.0	88%
	[*Sordaria macrospora*],					
	XP_003352871.1					
NCU09525	Bacterial-type extracellular	284	284	85%	2e–93	69%
	deoxyribonuclease [N. haematococc*a*],					
	XP_003051650.1					

**Figure 5:**
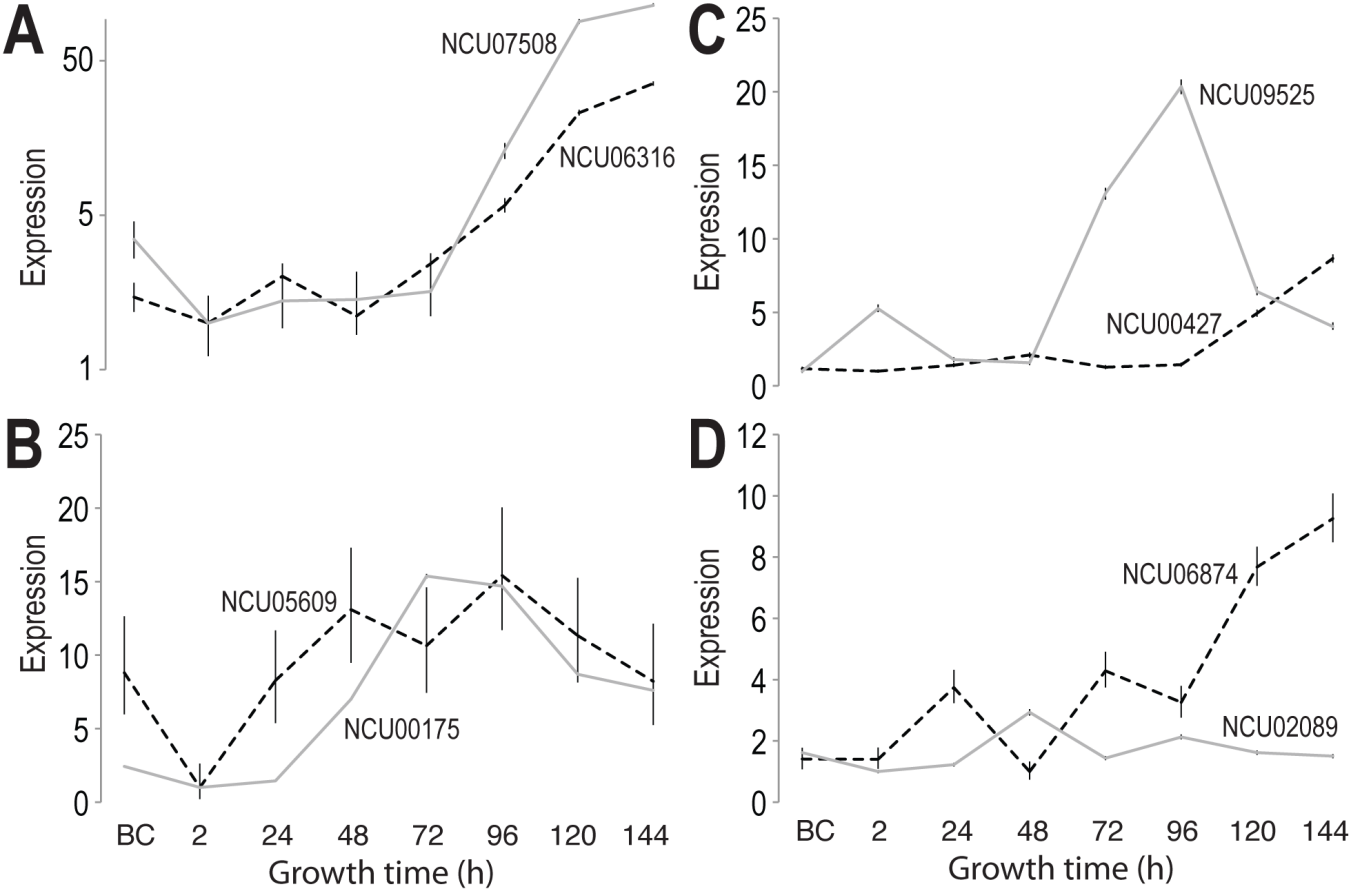
Expression patterns of eight genes with impact on successful perithecium formation in *N. crassa*. A. Expression of NCU06316 (solid line) and NCU07508 (dashed line), knockouts of which lead to early arrest of perithecial development at 48–72 h. Both genes exhibit a continuous increase of expression after 72 h. B. Expression of NCU00175 (solid line) and NCU05609 (dashed line), up-regulated during intermediate stages of perithecial development. C. Expression of NCU09525 (solid line) and NCU00427 (dashed line), both changing significantly in late perithecial development. D. Dynamic changes in expression of NCU02089 (solid line) and NCU06874 (dashed line) across perithecial development. Error bars indicate the inferred 95% credible interval.

The expression patterns of the genes whose knockouts showed an impact on fruiting body formation were monitored over the time course of sexual development starting from mature protoperithecia before crossing until 144 h, when wild-type cultures showed mature perithecia. The genes NCU06316 or 07508 exhibited a continuous increase in expression starting at 72 h (Fig. 5A). The increased expression correlated with the arrest of the fruiting body formation in an early stage between 48 and 72 h in deletion mutants.

Knockouts of NCU05609 (a proline-rich protein 8), 00175 (a repetitive proline-rich cell wall protein), 00427 (a YjeF_N domain-containing protein), 02089 (a hypothetical protein SMAC) and 09525 (a hypothetical protein SMAC) were arrested at the protoperithecium stage, indicating their importance in the formation of fruiting bodies. NCU09525 encodes a putative secreted protein and has been described in *Nectria haematococca* as bacterial-type extracellular deoxyribonuclease [63]. NCU09525 and 00175 were highly expressed between 48 and 120 h, while expression of NCU05609 peaked at 48 and 96 h after crossing. NCU06874 and 00427 showed an increased expression 96 h after crossing. NCU02089 peaked at 48 h after crossing and remained fairly constant across the remainder of sexual development (Fig. 5C–D). Although only protoperithecia were observed in mutant crosses, the culture medium for the knockout of NCU06874 blackened, indicating the secretion of melanin, and implying that melanin biosynthesis remained functional despite the fact that no perithecia were formed. Genes encoding enzymes in the melanin synthesis pathway showed a highly similar pattern across sexual development within each *Neurospora* species. While melanin synthesis genes were highly expressed in *N. crassa* protoperithecia samples, they were expressed at a lower level in *N. tetrasperma* and *N. discreta* protoperithecial samples than in late perithecial samples (Table S1). Further research is needed to understand function of melanin synthesis early sexual development in different *Neurospora* species. The protein encoded by NCU06874 contains a High Mobility Group (HMG) box, a structure often found in proteins involved in the regulation of DNA-dependent processes and DNA repair. Recent studies have demonstrated that deletion of the ortholog of NCU06874 in *Podospora anserina* and *Fusarium graminearum* had no effect on perithecium development [64], [65]. However, an effect on the distribution of perithecia in *P. anserina* was observed [64], [65].

The gene NCU02089 belongs to cluster 5. In *N. crassa*, this gene is expressed fairly uniformly, showing only a slight peak in expression at 48 h. The corresponding ortholog in *N. tetrasperma* shows a similar expression pattern. In *N. discreta*, in contrast, the expression of NCU02089 remains uniform until 96 h, then increases until the end of the sexual development.

The genes NCU06316, 07508, 05609, 00427, 09525, and 06874 belong to cluster 6. In *N. crassa*, the genes NCU06316 and 07508 are highly expressed after 72 h. In *N. tetrasperma*, as in *N. crassa*, the ortholog of NCU07508 also increases in expression after 72 h, while in *N. discreta* the expression peaks at 48 h, and increases again after 96 h. The expression of the ortholog NCU06316 in *N. crassa* differs from the one in *N. tetrasperma* and *N. discreta*. Over the time course of sexual development, expression of this gene is very low. The gene NCU05609 shows an increasing expression over the time course of the sexual development, peaking at 48 and 96 h. In contrast, the corresponding ortholog in *N. tetrasperma* does not show any changes in gene expression, while in *N. discreta* the expression decreases until 24 h, then remains steady until the end of the sexual development. In *N. crassa*, the expression of the gene NCU00427 remains constant until 96 h after crossing and then increases towards the end of the development. The expression of the corresponding ortholog in *N. tetrasperma* peaks at 24 and 120 h, while in *N. discreta*, this gene remains constant over the time course of fruiting body formation. The expression of the gene NCU09525 peaks between 48 and 120 h in *N. crassa*, while in *N. tetrasperma* and *N. discreta*, its expression peaks initially at 2 h, potentially as a consequence of mycelial disruption caused by the spreading of conidia and mating of the strains, and then increases until 96 h after crossing followed by a slight decrease towards the end of the development. The gene NCU06874 shows peaks in gene expression at 24 and 72 h, and an increase of expression from 96 h until the end of perithecium development. In contrast, in both *N. tetrasperma* and *N. discreta*, the expression of this gene remains fairly constant over the time course of the sexual development.

The gene NCU00175 belongs to cluster 8 and shows an increased gene expression between 24 and 120 h in *N. crassa*. A similar pattern was observed for *N. tetrasperma*, where the expression increased at 48 and 120 h. In *N. discreta* however, the gene expression increased steadily until 96 h after crossing, remained constant.

**Figure 6:**
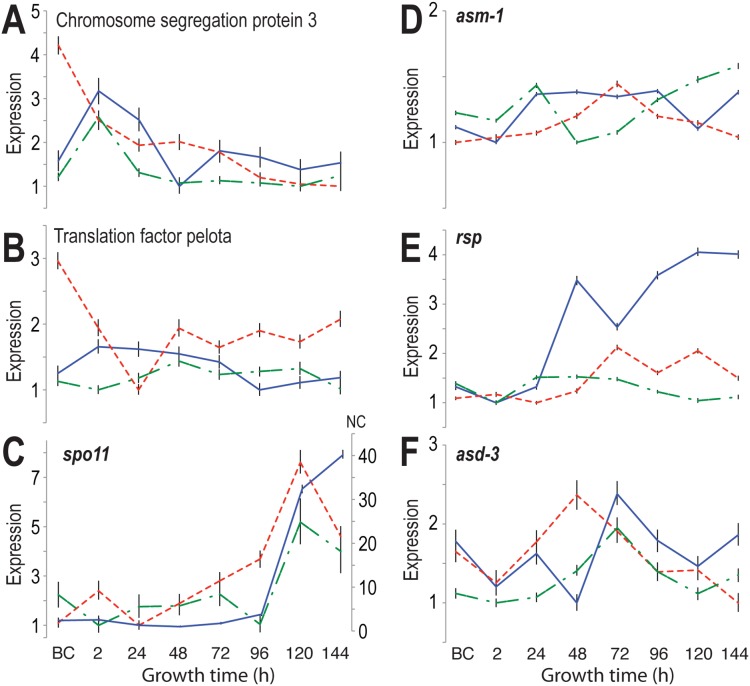
Comparative analysis of meiosis-related genes, exhibiting differences in gene expression between *N. tetrasperma* (red dashed line), *N. crassa* (blue solid line) and *N. discreta* (green dash-dotted line). A) Expression of the *N. crassa* gene encoding chromosome segregation protein 3 was up-regulated during early sexual development in both *N. crassa* and in the orthologous gene in *N. discreta*, but the *N. tetrasperma* ortholog was down-regulated across sexual development, B) Expression of the gene encoding translation factor pelota in *N. crassa* and expression of its ortholog in *N. discreta* was consistent, but the ortholog in *N. tetrasperma* was dramatically and dynamically differentially expressed. C) Expression of meiosis specific gene *spo-11* and its orthologs was up-regulated for all three species from 96 h after crossing, but the up-regulation started early for the *N. tetrasperma* ortholog. D) Expression of *asm-1* (ascus maturation) in *N. crassa* and its orthologin *N. discreta* exhibited the same two-peaked pattern, featuring a peak of expression during early development of the perithecium and a second peak at a later stage of perithecial development, but the ortholog in *N. tetrasperma* exhibited only one peak 72 h after crossing. E) Expression of *N. crassa rsp* (round spore) and its ortholog in *N. discreta* exhibited up-regulation preceding 48 h, but upregulation in *N. tetrasperma* preceded 72 h. F). Expression of *N. crassa asd-3* (ascospore development) peaked at 72 h in *N. crassa,* as did its ortholog in *N. discreta*, but expression of the ortholog in *N. tetrasperma* peaked at 48 h. Error bars indicate the inferred 95% credible interval.

Key differences in gene expression patterns of meiosis-related genes between *N. tetrasperma* and *N. crassa* as well as *N. discreta* were observed (Fig. 6). Many RNA processing genes and meiosis- and mitosis- specific genes have experienced multiple duplications. Only a few single copy meiosis-specific gene homologs have been confirmed with our methods. Among these genes, we observed differences in gene expression between homothallic *N. tetrasperma* and heterothallic *N. crassa* and *N. discreta*. The meiotic chromosome segregation protein 3 homologs, *N. tetrasperma* Neute1draft_73667 (JGI identifier for the *mat A* genome), *N. crassa* NCU01858 (Broad Identifier), and *N. discreta* Ndisc8579_91937 (JGI identifier) showed a consistent gene expression pattern in *N. crassa* and *N. discreta*: two up-regulations at 2 h and 72 h (Fig. 6A). In contrast, the expression of the ortholog of chromosome segregation protein 3 in *N. tetrasperma* exhibited a steep down-regulation after strains were crossed and only a slight up-regulation 24 h after crossing. Furthermore, the translation factor pelota (Neute1draft_80736, NCU09161, and Ndisc8579_129376), which functions in meiotic cell division, exhibited a generally consistent expression across perithecial development in *N. crassa* and *N. discreta*, but a dynamic change in expression in *N. tetrasperma* during early perithecial development (Fig. 6B). A dramatic up-regulated expression of the meiosis-specific gene *spo11* (Neute1draft_98113 m, NCU01120, Ndisc8579_51404) started early in *N. tetrasperma* 24 h after crossing, but began in *N. crassa* and *N. discreta* 96 h after crossing (Fig. 6C). For sporulation-related genes, including *asm-1* (ascospore maturation-1), Neute1draft_115825, NCU01414, Ndisc8579_123166), *Rsp* (round spore, Neute1draft_126958, NCU02764, Ndisc8579_94666), *asd-3* (ascus development-3, Neute1draft_103712, NCU05597, Ndisc8579_126512), we also observed up-regulation of expression at the same stages between *N. crassa* and *N. discreta* (Fig. 6D–F). Up-regulation of homologs of *N. crassa asm-1* and *rsp* in *N. tetrasperma* occurred later than in *N. crassa* and *N. discreta*, but interestingly the homolog of *N. crassa asd-3* exhibited an early up-regulation for *N. tetrasperma* at 48 h. Similar up-regulation of *asd-3* for *N. crassa* and its homolog in *N. discreta* occurred at 72 h after crossing. The genes NCU01858 and 09161 belong to cluster 1, the genes NCU01120, 02764 and 05597 to cluster 6 and NCU01414 to cluster 7. Except NCU01858 and 09161, all genes shown in Fig. 6 belong to the candidate list.

### 3.4 Phenotypes of seven knockout mutants cosegregate with hygromycin resistance

To validate the linkage between the insertional mutation and the phenotype, we followed a strategy developed previously for *N. crassa* KO strains [56], and backcrossed progeny (ascospores) were selected and germinated. Twelve to twenty single ascospore progeny displayed hygromycin resistance, and complete cosegregation of hygromycin resistance and identified phenotypes was observed for seven out of eight investigated genes (Table S6). The exception was the knockout of NCU09525, for which only a KO strain of *mat A* was available. No perithecia were produced in this mutant-wild type cross, preventing us from assessing segregation of the cross.

## Discussion

Here, we compared the sexual development of three closely related *Neurospora* species, *N. crassa*, *N. tetrasperma*, and *N. discreta,* focusing on characterizing expression patterns for genes involved in sexual development and identifying new genes or new functions of annotated genes in regulating sexual reproduction, taking advantages of the distinct differences among these otherwise highly similar and closely related species. The comparison of gene expression levels across these species during fruiting body formation revealed eight genes that were shown to be crucial for the successful development of perithecia in *N. crassa*: their knockouts were unable to produce mature perithecia, and seven of the eight exhibited cosegregation of the phenotype and a hygromycin marker. We identified different expression patterns for meiosis-related genes between *N. tetrasperma* and *N. crassa* and *N. discreta* that correspond with observed differences in meiosis and sexual spore development between pseudohomothallic *N. tetrasperma* and heterothallic *N. crassa* and *N. discreta*. Since meiosis gene sets and sporulation machinery are largely conserved in presence as well as sequence within these genomes, consistent differences among functionally related genes in these species would be strong evidence of dependent associations for the reconstruction of gene networks and thus for understanding the genetic basis of meiosis and sexual sporulation in *Neurospora* and ascomycetes. Additionally, species-specific expression in later stages of development was observed when clustering the ortholog genes. Future efforts should expand analysis to identify a more complete set of genes involved in meiosis and sporulation based on genetics and reverse genetics on the model *N. crassa*.

We distinguished eight gene clusters based on similarity of overall expression patterns across sexual development in three *Neurospora* species. During early sexual development, genes involved in metabolism are enriched, and clusters that appear later during sexual development are enriched with genes involved in metabolism, energy, transcription, cell cycle, and DNA processing. The expression profiles of these clusters correlated with the morphological changes observed during sexual development: at first metabolic genes are upregulated, then transcription and cell cycle related genes are activated, and eventually protein synthesis and transport genes are transcribed to deliver proteins needed to their destination in order to form the complex three dimensional fruiting body.

Our study revealed eight genes that are required for the successful formation of fruiting bodies in *N. crassa*. Knockouts of two genes, NCU06316 and 07508 resulted in the arrest of perithecium formation between 48 and 72 h after crossing (Fig. 4, panels B and C). In correlation with the gene expression data (Fig. 6A), these genes are of great importance in the later stage of development when structures such as the perithecial beak or ascospores are developed. The developing perithecia for NCU06316 (a putative argonaute siRNA chaperone complex subunit) and 07508 (a putative type-2 protein geranylgeranyltransferase subunit) remain round-shaped without formation of a beak. Six crosses of *N. crassa* mutant strains showed only protoperithecia and no formation of fruiting bodies. These results correlate with the gene expression observed over the time course of the sexual development in *N. crassa*. Here, an up-regulation of gene expression during the later stages of the development was observed. Our observations indicate that there are several genes that are required for the successful formation of perithecia.

## Conclusions

We have used comparative transcriptomics as a tool to identify genes that are required for the successful formation of fruiting bodies. The impact of the deletion of the candidate genes was demonstrated in phenotypes observed in crosses exhibiting impaired perithecium formation at different stages of the development. Our findings shed light on the developmental process of fruiting body formation, evolution, and spore development in *Neurospora* species, thus establishing the foundation for future research particularly related to closely related pathogenic fungi. We suggest the potential utility of future research on the eight genes that we have discovered as essential contributors to fruit body development and as candidate genes for targets in the development of fungicides for control of plant pathogens.

## Supporting Information

Table S1 **Expression results of all genes of all three Neurospora species.**
(XLSX)

Table S2 **Level of Expression (LOX) data of** ***N. crassa, N. tetrasperma*** **and** ***N. discreta*** **orthologues.** LOX estimates the Level Of gene eXpression from high-throughput-expressed sequence datasets with multiple treatments or samples. The tables includes the corresponding upper and lower confidence intervals (CI) across developmental stages.
(XLSX)

Table S3 **Comparative gene expression analysis of** ***N. crassa, N. tetrasperma,*** **and** ***N. discreta*** **.** The table includes the results from the calculations such as the differences of confidence intervals.
(XLSX)

Table S4 **Comparison of species using the IF statement function.** In each comparison the highest value across all time points for each gene was selected (MAX) and the maximum values were then sorted in descending order.
(XLSX)

Table S5 **Prioritization of the maximum value across the time course of the sexual development for each gene in speecies comparisons.**
(XLSX)

Table S6 **Cosegregation of hygromycin resistance and identified phenotypes in KO strains of interest.**
(DOCX)
