# Supplementary material for: Gene Expression Differences among Three Neurospora Species Reveal Genes Required for Sexual Reproduction in Neurospora crassa
Source: PLoS One. 2014 Oct 20;9(10):e110398. doi: 10.1371/journal.pone.0110398 (PMC4203796; doi:10.1371/journal.pone.0110398)
Supplement: Table S6 — Cosegregation of hygromycin resistance and identified phenotypes in KO strains of interest. (DOCX) [file pone.0110398.s006.docx]

**Table S6:** Cosegregation of hygromycin resistance and identified phenotypes in KO strains of interest.

| Gene | strains  (FGSC) | phenotypes | Co-segregation | |
| --- | --- | --- | --- | --- |
|  |  |  | ascospores | Phenotype/hyg^+^ |
| NCU00175 | 12193a  12194A | Protoperithecia, few peritehcia  arrest in very early developing stage. | Only in KO *mat-a*  x wildtype *mat-A* | 12/12 |
| NCU00427 | 13288a  13289A | Protoperithecia | normal | 16/16 |
| NCU02089 | 19087a  19088A | Protoperithecia, few peritehcia  arrested in very early developing stage | Only in KO *mat-a*  x wildtype *mat-A* | 20/20 |
| NCU05609 | 18207A  18208a | Protoperithecia, few peritehcia  arrested in very early developing stage | Only in KO *mat-a*  x wildtype *mat-A* | 17/17 |
| NCU06316 | 20345a  20346A | Perithecia arrested at early stage | normal | 12/12 |
| NCU06874 | 20212a  20213A | Protoperithecia, dark pigment in medium | normal | 12/14* |
| NCU07508 | 18104A  18105a | Perithecia arrested at early stage | normal | 14/14 |
| NCU09525 | 21153A | Protoperithecia | no | N/A |

*Pigments production was variable in quantity, and two hygromycin resistant strains showed no obvious dark pigments in SCM medium.
